# The role of forensic imaging in the allegations of torture in asylum seekers

**DOI:** 10.1007/s00414-025-03601-3

**Published:** 2025-09-18

**Authors:** Giuseppe Davide Albano, Giuseppe Lo Re, Sergio Salerno, Marika Barberi Triskari, Mariagrazia Fornasari, Giuseppe Micci, Domenico Albano, Mauro Midiri, Corinne La Spina, Ginevra Malta, Stefania Zerbo, Antonina Argo

**Affiliations:** 1 Department of Health Promotion, Mother and Child Care, Internal Medicine and Medical Specialties, Institute of Legal Medicine, University of Palermo, Palermo, Italy; 2 Department of Biomedicine, Neuroscience and Advanced Diagnostics, Radiology Department, University of Palermo, Palermo, Italy; 3 Department of Radiology, ASST Grande Ospedale Metropolitano Niguarda, Milan, 20161 Italy; 4 Dipartimento di Scienze Biomediche, Chirurgiche ed Odontoiatriche, Università degli Studi di Milano, Milan, Italy; 5 Department of Health Promotion, Mother and Child Care, Internal Medicine and Medical Specialties, Institute of Legal Medicine, University of Palermo, Via del Vespro 129, Palermo, 90100 Italy

**Keywords:** Forensic imaging, Radiology, Torture, Mistreatment, Asylum seekers, Injury, Istanbul protocol

## Abstract

Nowadays, according to the Istanbul Protocol, diagnostic tests are not an essential part of the clinical assessment of a person alleging torture or ill-treatment. In many cases, a medical history and physical examination are sufficient. However, literature evidence suggests a helpful role of diagnostic imaging in the allegation of evidence of torture, especially in the case of musculoskeletal injuries. The purpose of this narrative review is to highlight the role of imaging tests in suspected torture victims, emphasizing the role of these methods in establishing legal evidence of mistreatment. No specific imaging features are described in the literature, however, musculoskeletal imaging allows the detection of previous fractures, bone deformities, and tendon and ligament injuries. These are mainly due to blunt force injuries. MRI is the most helpful imaging tests to allegate evidence of Falaka. Imaging tests, particularly conventional X-rays and CT, are helpful in forensic investigations of when there is suspicion of retained foreign bodies, such as bullets or shrapnel, especially in cases where clinical examination is inconclusive. CT and MRI can show late sequelae of head trauma such as subdural hematomas, hygromas, old intracerebral bleeding, and hydrocephalus. The current literature highlights the importance of subjecting patients to imaging and specialists examinations to document evidence of torture and support the assessment of the degree of consistency with the reported history. These are second-level investigations that must be targeted to specific diagnostic questions and preceded by a thorough examination conducted by experts in the field using standardized methodologies, in line with the recommendations of the Istanbul Protocol. Imaging studies can bridge the gap between clinical examination and the patient’s history.

## Introduction

Torture is the intentional, organized, or reckless imposition of physical or psychological distress by either an individual or a group acting independently or under the authority of a higher entity [1]. This is done to coerce another individual into revealing information, eliciting a confession, or for various other motives. Establishing definite proof of an alleged torture incident presents challenges for forensic investigators, especially when dealing with individuals evaluated several months after the physical trauma [2, 3]. The Istanbul Protocol, a set of recommendations established by the United Nations for healthcare professionals aiding individuals who have endured torture, offers instructions on the assessment and assessment processes concerning particular torture techniques, including but not limited to physical assaults, blunt force injuries, burn injuries, dental harm, and sexual violence [4].

Nowadays, according to the Istanbul Protocol, diagnostic tests are not an essential part of the clinical assessment of a person alleging torture or ill-treatment. In many cases, a medical history and physical examination are sufficient. However, literature evidence suggests a helpful role of diagnostic imaging in the allegation of evidence of torture, especially in the case of musculoskeletal injuries [5–7]. Nevertheless, due to the lack of availability in all contests, the cost and availability of such procedures prevents widespread utilization [5]. Moreover, the use of imaging on victims of torture may have an emotional and psychological impact on patients and rises important ethical issues, limiting the use of time-consuming and invasive investigations [8, 9].

Sicily is a destination country for many people and is a frequent recipient region of migrants in the Mediterranean and southern Italy [10–12]. The Institute of Forensic Medicine at the University of Palermo, in collaboration with the University of Palermo, the Radiology Department and local non-profit organizations like Médecine Sans Frontières, conducts medico-legal assessments of torture victims to offer expertise, standardized methodologies, and impartiality in support of asylum applications. Our objective is to provide verifiable evidence of torture to local commissions by the principles outlined in the Istanbul Protocol. Furthermore, certain cases necessitate a multidisciplinary approach to reinforce the proof of torture [13, 14].

Our examinations involve a multidisciplinary team consisting of a forensic physician specialized in identifying signs of torture, a psychologist, a resident doctor in Forensic Medicine, a cultural mediator, and, when indicated by the type of trauma or clinical findings, consultations with relevant medical specialists—such as ophthalmologists, otorhinolaryngologists, or forensic odontologists—to further assess injury consistency, evaluate potential long-term consequences, and conduct additional clinical, imaging, or diagnostic tests, with the patient’s consent. The purpose of this narrative review is to highlight the role of imaging tests in suspected torture victims, emphasizing the role of these methods in establishing legal evidence of mistreatment.

## Methods

We performed an unsystematic narrative review of the existing literature, utilizing databases such as PubMed and Scopus [15]. Initially, our search was restricted to peer-reviewed original articles, case report and case series, identified through searches of abstracts and titles using keywords such as “forensic imaging”, “radiology”, “torture”, “asylum seekers”, “ill-treatment”, “injury”, from 1990 to 2024. Two researchers independently screened the articles, eliminating the duplicates and comparing the findings. In case of discordance a third researcher has been consulted. We included studies focusing on developing, using, and commenting the use of imaging techniques in approaching victims of torture/mistreatment and asylum seekers for identification of injuries in forensics, with no restrictive inclusion criteria about the type of the paper. Moreover, we included relevant studies that evaluated the impact of forensic imaging in the evaluation of victims of trauma in forensics. Articles not in English, commentaries, abstracts, posters, conference proceedings were excluded. To augment our findings, we conducted additional searches using Google Scholar and the Google Search engine to access grey literature, including sources relevant to this study’s highlighted examples. All eligible studies published before January 1 st, 2025 were collected. For each article, the reference list was manually checked in order to identify any additional relevant paper which may have not been found in the initial search.

## Results

### Musculoskeletal injuries

One of the most challenging issues of clinical forensic medicine applied to asylum seekers who are victims of torture is the excessive time interval between the violence suffered and the clinical examination. Skin injuries only or no macroscopic signs of injury can be present at inspection [3, 16, 17]. Musculoskeletal imaging leads to the detection of trauma signs and their consistency with violence, supporting the final judgment on whether the outcomes are attributable to torture, in accordance with the Istanbul Protocol [5, 18]. No specific imaging features are described in the literature, however, musculoskeletal imaging allows the detection of previous fractures, bone deformities, and tendon and ligament injuries. These are mainly due to blunt force injuries [5, 18]. This evaluation should be preceded by a clinical and medical history assessment to understand the dynamics of the injury, pre-existing medical conditions and previous traumas that may affect imaging features and causality assessments.

Bone scintigraphy permits the detection of trauma, even in the absence of radiographic signs on conventional radiology. It is a nuclear imaging examination whose principle lies in the link between technetium-based contrast and osteoblasts that demonstrate activity upon trauma [19]. Lok et al. showed that bone scintigraphy Can remain positive for up to 5 months, also showing periosteal damage following trauma [20]. However, it is an invasive test that requires bioethical and appropriateness evaluations to be used to ascertain torture outcomes, considering the amount of radiation and the absence of a morphological but only functional data.

Ultrasound is a minimally invasive operator-dependent technique that does not allow objective preservation of images for a second-opinion or for alleging violence and torture signs. It can be useful for tendinous and ligamentous injuries but not diriment to evaluate fracture outcomes and bone deformities [5, 18, 19].

Magnetic resonance imaging (MRI) is the most helpful method in cases of tendon, muscle, and ligament injuries. MRI can be used to identify direct and indirect signs of recent or chronic tears, detecting edema of bone and soft tissues, effusion, collections, fibrosis and scars [4]. MRI is a helpful tool also in specific type of fractures (tarsus, elbow, scaphoid), especially in the pediatric patient, but within a narrow time range [4, 21]. In addition, T1 and T2 values may change in relation to the healing stage of a fracture, providing useful information about the consistency with more or less recent trauma [22]. With the exception of falaka, there are no studies in the literature that have validated MRI as a tool for detecting torture in the living.

**Fig. 1:**
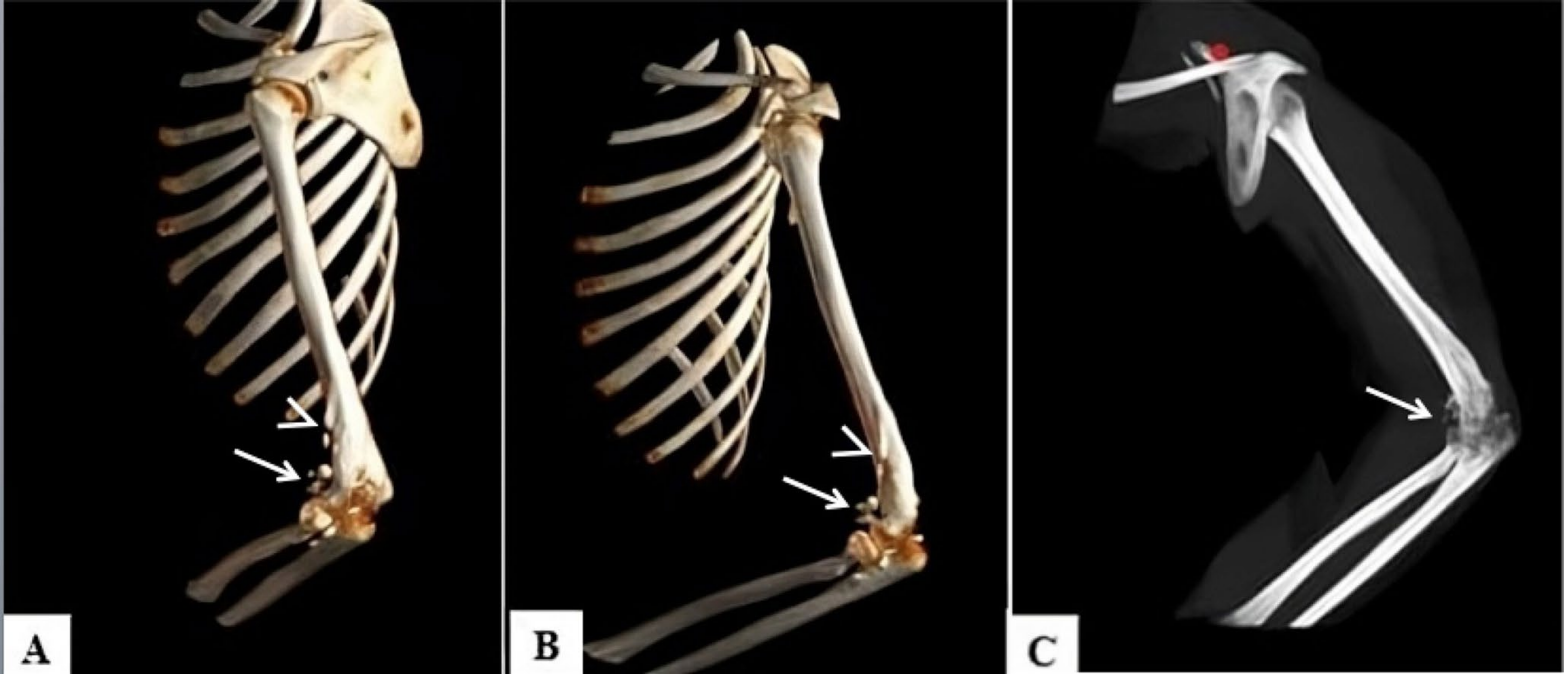
Victim of torture due to blunt force injuries in the upper limb and signs of deformation. CT with volume rendering reconstructions (lateral projection **A**, anteroposterior projection **B**) and maximun intensity projection (anteroposterior projection **C**) shows: structural deformity as results of complex fracture with alteration of the mid-distal third of the humerus, the humeral epicondyles, the ulnar olecranon, and the proximal epiphysis of the radius (especially the coronoid process); bone fragments coexist in the context of the surrounding soft tissues (white arrow). Also note marginal osteophytosis likely due to a post-traumatic arthritic process on a degenerative basis (white arrowhead)

**Fig. 2:**
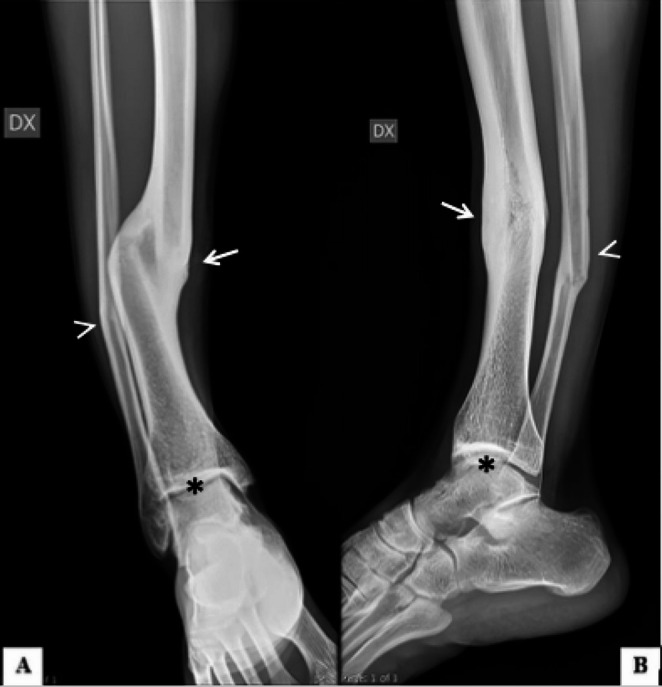
Victim of torture due to blunt force injuries in the lower limbs and signs of deformation. Leg x-ray in anteroposterior (**A**) and lateral (**B**) projections shows results of displaced fracture affecting the distal part of the right (DX) tibial (white arrow) and fibular diaphysis (white arrowhead) with residual structural deformity. Also note mild degenerative changes of the tibiotalar joint with reduction of the joint space and osteophytes (black asterisk)

Computed tomography (CT) offers a detailed view of fracture and bone deformities. It also provides through Volume rendering and multiparametric reconstruction methods valuable visual support to allege intentional violence and torture signs (Figs. 1). Radiography also allows the detection of fracture and bone deformation outcomes while exposing the patient to a much lower dose of ionizing radiation [5, 18] (Fig. 2).

Thus, musculoskeletal imaging can provide information about the post-traumatic time interval (PTTI), which depends on the bone’s response to the healing process after trauma. A recent study performed on 545 radiographs from 212 tubular fractures in 162 adult patients showed that fracture healing time and imaging features assessed according to the adapted fracture healing scale (AFHS) are conditioned by sociodemographic factors and clinical characteristics of the patient (fracture type and surgical approach). In addition, a linear correlation between PTTI and AFHS has been demonstrated, showing a progression over time of radiological signs as well due to injury [23]. Imaging may therefore play a crucial role in attaching evidence of torture and mistreatment, increasing the robustness of the consistency judgment of the clinical forensic examination.

### Falaka

Falaka is a distinctive form of torture characterized by repeated blunt-force trauma to the soles of the feet. It is one of the most frequently reported methods of torture, particularly in the Middle East. Acutely, it can result in foot swelling, hematomas, lacerated-contused wounds of the skin, functional impairment, and difficulty walking [24, 19]. In the long term, diagnosis can be challenging, as specific external signs of previous falaka may be absent. However, the lack of external signs does not rule out its occurrence. Imaging techniques can serve as a valuable tool in identifying falaka-related injuries and assessing their compatibility with reported events [24, 19].

Bone scintigraphy may be useful in forensic investigations. Falaka, due to fractures and bone lacerations, can induce a periosteal reaction—sometimes irreversible—that is not detectable through clinical examination or conventional radiography. However, bone scintigraphy Can reveal such changes even up to 12 years after the traumatic event [19, 25]. A case reported by Altun et al. showed an increased blood flow in the feet and distal tibia after falaka injury with no MRI findings in plantar structures, due to bone trauma [26].

**Fig. 3:**
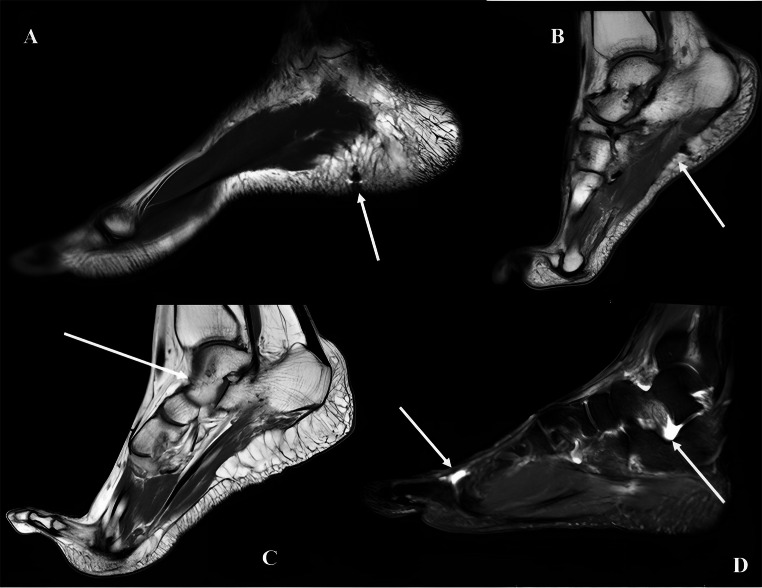
MRI of the foot findings of four subjects with clinical signs and history suggestive for previous falaka. (**A**) Sagittal TSE T2-weighted image of the right foot: thickening of the fatty tissue of the heel with irregular distribution of the fatty fibrous septa (thinned or absent) with an hypointense image in the subcutaneous area of the heel responsible for magnetic susceptibility artifacts who refers likely to a foreign body (white arrow). (**B**) Sagittal TSE T1-weighted image of the right foot: thinning of the plantar fascia; slight thickening of the fatty tissue of the heel with irregular distribution of the fatty fibrous septa (thinned or absent) with an hypointense area who refers likely to fibrosis (white arrow); (**C**) Sagittal T1-weighted image of the right foot: severe thinning of the plantar fascia; significant thickening of the fatty tissue of the heel with irregular distribution of the fatty fibrous septa (thinned or absent); irregularity of the dorsal cortex of the talus bone (white arrow). (**D**) Sagittal STIR image of the right foot: thin fluid collect on the dorsal side of the 1 st metatarsophalangeal joint and under the Talus bone (white arrows)

As suggested by the Istanbul Protocol, the injuries are often localized in the soft tissues. In this regard, MRI is the most helpful imaging tests to allegate evidence of Falaka. However, the clinical examination is still the diagnostic method in the acute phase of the injury [4]. Savnik et al. evaluated the usefulness of MRI of the foot in 12 victims of Falaka highlighting his role to prove the falaka in torture victims. Typical observed MRI features were thickening of the plantar aponeurosis, signs of inhomogeneous aponeurosis, periosteal irregularity [27] (Fig. 3). Plain radiography and CT can be used for detecting bony deformities due to previous fractures of the tarsus and metatarsal bones of victims of falaka.

### Foreign body retention

Imaging tests, particularly conventional X-rays and CT, are helpful in forensic investigations of when there is suspicion of retained foreign bodies, such as bullets or shrapnel, especially in cases where clinical examination is inconclusive [28, 29]. Gunshot wounds, in particular, may leave minimal or no external evidence if the entry wound is small, heals over time, or if the projectile remains embedded deep within tissues.

Conventional X-rays are the first-line imaging modality for detecting metallic foreign bodies, such as bullets, fragments, or metallic debris, bone fragments, which appear as high-density objects. It is noninvasive, inexpensive and widely available, therefore it is the first choice to screen the presence of a foreign body or fragment [30]. CT scans provide superior resolution and can identify deeply embedded foreign objects, bone fractures or fragments, or secondary complications such as infections, abscesses, or chronic inflammatory changes [30]. CT and three dimensional reconstructions enhance the localization of projectiles and their trajectory, crucial for forensic documentation [18, 19, 30]. MRI provides the highest accuracy to evaluate the foreign body retention long-term effects, such as soft tissue abscess, osteomyelitis, septic arthritis [30].

**Fig. 4:**
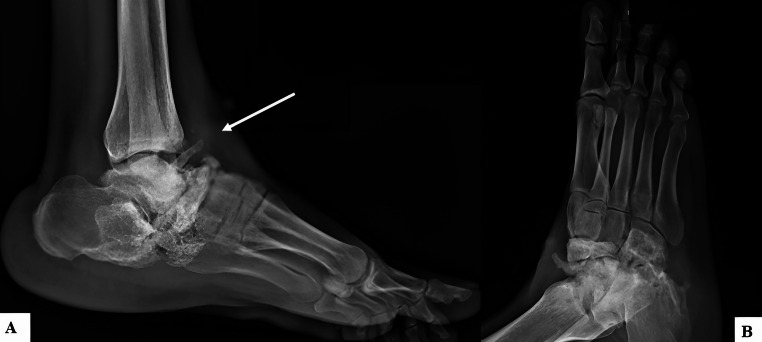
Ankle and foot X-ray of an asylum seeker victim of gunshot wound. Xray in anteroposterior (**A**) and lateral (**B**) projection shows foreign body retention due to gunshot wounds in the subcutaneous tissue anterior to the Talus bone (white arrow)

When clinical findings are absent or ambiguous, imaging could serve as an objective tool to corroborate the presence of direct or indirect signs of foreign body retention due to torture (Fig. 4).

### Head trauma

Brain damage due to torture can be related to several mechanisms: direct effect of trauma more commonly, effects of poisoning, malnutrition, starvation, asphyxiation, hypoxia, neuropsychiatric sequelae [4]. Head trauma is the leading cause of torture and maltreatment. In the acute phase scalp bruises and laceration can be observed. After healing scars are most frequent observed injuries [31, 32]. CT and MRI can show late sequelae of head trauma such as subdural hematomas, hygromas, old intracerebral bleeding, and hydrocephalus [6].

A recent case series described the forensic approach on asylum seekers victims of torture with a history of traumatic brain injury (TBI). The authors described three main symptoms domains in case of TBI: somatic symptoms (headache, sleep disorders, vestibular impairment), cognitive disfunctions, affective disorders. In one of the case the patient suffered from epilepsy due to previous head trauma and underwent brain MRI that showed “encephalomalacia and gliosis in the bilateral frontal lobes, suggestive of brain injury, atrophy, and hemorrhage consistent with the reported prior assault” [33]. In case of dizziness or ear loss, a second level evaluation could include CT or MRI, together with clinical and other diagnostic specific tests, to examine fractures of the temporal bone or the involvement of ear ossicular chain [4].

**Fig. 5:**
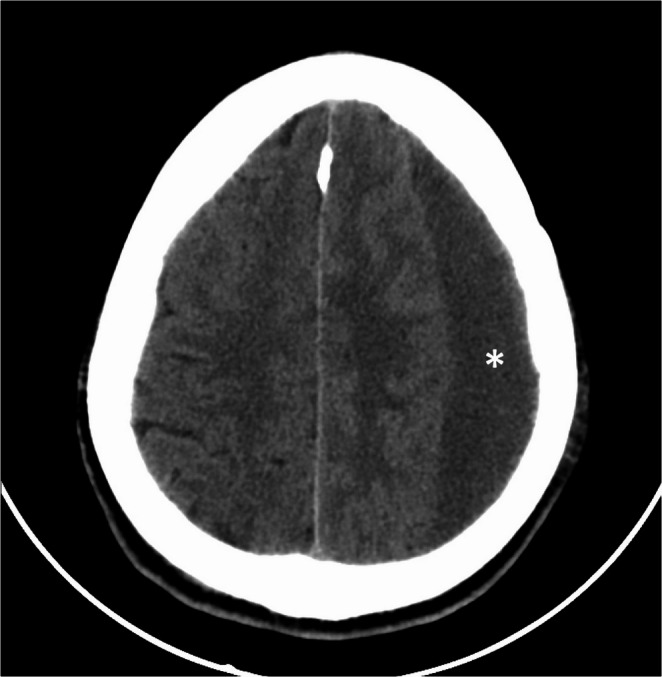
A 36-year-old male migrant, reported history of violence with head trauma. Axial CT of head: chronic subdural hematoma in the left hemisphere (asterisk)

Torture victims examination could require neuropsychological assessment to document post-traumatic stress disorder (PTSD). PTSD is a psychiatric disorder with no visible brain lesions in some cases [4]. In their observational study Zandieh et al. evaluated the Metabolic and Structural brain changes in patients with Torture related PTSD, by using MRI and 18F-FDG-PET. They demonstrated that subjects with severe PTSD typically exhibit elevated symptom levels, including heightened arousal, increased vigilance, sleep disturbances, and impulsive outbursts. Alterations in the body’s biological stress response may persist for years following the initial trauma. This study investigates metabolic and structural brain changes in individuals with and without treatment-resistant PTSD (TR-PTSD). Notably, structural brain differences in patients with TR-PTSD were most pronounced in the hippocampus, as suggested by previous literature [34, 35] (Fig. 5).

### Dental trauma

**Fig. 6:**
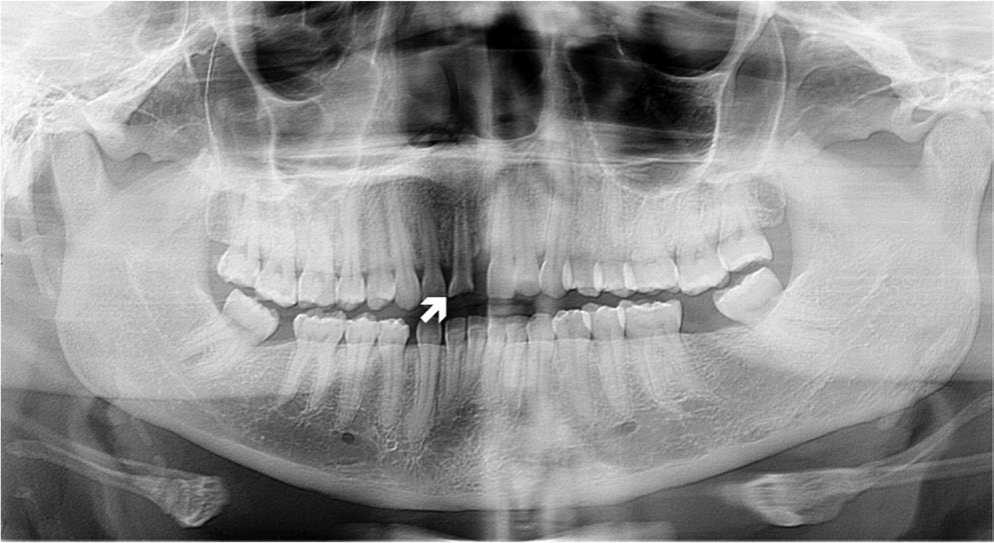
Orthopantomography of a 28-year-old male migrant, history of dental trauma. Fracture of an upper central incisor (arrow). Also note the loss of both lower second molars

Dental cavity need to be examined in victims of torture. Poor quality detention conditions can impair oral health or worsen previous conditions. Moreover, direct trauma, thermal injuries, electric injuries can cause oral lesions such as tooth avulsions, teeth fractures, dislocations, mucosal injuries. Imaging diagnostic tests may be helpful to detect soft tissue, temporo-mandibular or dental trauma evidence [4]. The literature suggests the importance of the involvement of a forensic odontologist in these cases, to confirm suspected cases of injury due violence and abuse [36]. Crosby et al. described in their work the prevalence and methods of torture and its sequalae in head and neck district [37]. Application of blunt force injury to the head and neck can lead to facial fractures, lacerations of the skin and consequent scars, timpanic membrane rupture, temporomandibular joint disorders [37, 38] (Fig. 6).

### Other injuries

Several methods of torture are described in the literature: blunt force injuries, postural torture, sharp force injuries, compression, thermal injuries, electric torture, dental torture, water and food deprivation, forced ingestion, sexual abuse [4, 5, 12, 39]. All these methods can lead to acute and long-term sequelae that can be object of imaging tests evaluation, thereby it is advisable to supplement the medicolegal report with other diagnostic imaging tests or specialist consultation if available [40, 41]. Neufeld et al. described the findings of a series of forensic evaluation of survivors of victims of torture with restraint/handcuff injuries. Handcuff use can cause fractures of wrist bones that can be highlighted by conventional X-ray [41]. MRI is the preferred method in case of positional torture, due to the involvement of soft tissues, tendons and ligaments. Sexual abuse can cause genitourinary injuries, in this case ultrasound can be a helpful and not invasive required imaging test [4].

The application of forensic imaging techniques for the documentation of evidence of torture is summarized in Table 1.

**Table 1:** Summary of the application of imaging technique to highlight evidence of torture in asylum seekers. (PTTI: post-traumatic time interval; PTSD: post-traumatic stress disorder; OPT: orthopantomography)

Muscoloskeletal Injuries	• *X-rays*: fractures, deformations, PTTI. • *MRI*: tendon, ligament, muscles, vertebral disc injuries and soft tissues. • *CT*: small fractures, deformations, PTTI. • *Ultrasound*: only for recent trauma (subcutaneous ecchymosis). • *Bone scintigraphy*: periosteal damage, and evidence of increased blood flow for a longer time after trauma.
Falaka	• *MRI*: Thickening of plantar fascia, bone bruise, oedema. • *CT*: fractures of tarsal and metatarsal bones. • *Bone scintigraphy*: signs of increased blood flow for a longer time after trauma.
Foreign body retention	• *X-rays*: first choice exam for metallic foreign body. • *CT*: superior resolution, bone fragments, foreign body complications (abscesses, chronic inflammation). • *MRI*: soft tissues infections, osteomyelitis, osteoarthritis.
Head trauma	• *CT and MRI*: intracerebral and subarachnoid hemorrhage, subdural and epidural hematoma, gliosis, hygroma, atrophy. • *PET*: altered metabolism due to PTSD.
Dental trauma	• *OPT*: dental age, teeth avulsion and fracture. • *CT*: superior resolutions, trauma and poor hygiene and health consequences. • *MRI*: temporomandibular joint disorder.
Other Injuries	• *X-rays*: wrist fractures in case of ligature. • *MRI*: soft tissue and nerves involvement in positional torture. • *Ultrasound*: genito-urinary complications.

## Discussion

The current literature highlights the importance of subjecting patients to imaging and specialists examinations to document evidence of torture and support the assessment of the degree of consistency with the reported history. These are second-level investigations that must be targeted to specific diagnostic questions and preceded by a thorough examination conducted by experts in the field using standardized methodologies, in line with the recommendations of the Istanbul Protocol. Imaging studies can bridge the gap between clinical examination and the patient’s history. The most frequently observed injuries are scars; however, the long-term effects of musculoskeletal injuries, head trauma, and various forms of torture and mistreatment can also be detected even years later. The signs of torture are often healed at the time of examination and the absence of scars does not exclude a previous torture [6, 42, 43].

Diagnostic imaging, when combined with a thorough clinical examination, can be a valuable screening tool for the early identification of signs of violence and torture in immigrant populations. Given the challenges in recognizing physical and psychological trauma in asylum seekers and migrants, integrating imaging techniques can help detect injuries that may not be evident during standard medical evaluations. Radiological assessments can reveal musculoskeletal damage, fractures, head trauma, and other sequelae of ill-treatment, providing objective documentation that supports clinical findings. X-ray is the first line method for the diagnosis of fractures and its long term effects. However, CT and MRI are preferred in case of small and spine fractures. For tendons, joints, inner organs and cavities ultrasound is the first choice. CT and MRI offer much more support, allowing the diagnosis of hematomas, tears and inflammation signs in soft tissues. Scintigraphy may be helpful to provide evidence of long lasting inflammation and degenerative changes [44]. A recent case series evaluated the role of functional imaging to implement clinical forensic examination in case of trauma. Functional disturbances can be more suggestive than anatomical abnormalities in some cases, providing additional impact on functional consequences [45]. Moreover, a recent study demonstrated a correlation between the detection rate of a bone lesion on scintigraphy and the time duration of torture [25].

As a first-line screening approach, this combined evaluation can guide healthcare professionals in determining the need for further specialized assessments, preferably in specialized referral centers. In case of unaccompanied minors imaging techniques (X-ray, orthopantomograms and CT), can be used to analyze some areas that can provide information for age estimation (hand-wrist, clavicle, teeth) [46–48]. In this regard, in cases where X-ray examinations are legally permissible despite the absence of medical necessity, the German Study Group on Forensic Age Diagnostics (AGFAD) provides a highly regarded, standardized protocol for age assessment. This approach underscores the importance of combining a physical examination and medical history (anamnesis) with an X-ray of the hand and a dental assessment, including evaluation of an orthopantomogram. If the hand skeleton has fully matured, the protocol further recommends a CT scan of the clavicles to enhance accuracy in determining age in adolescents and young adults [49, 50]. When findings suggest a history of violence or torture, second-level examinations in dedicated forensic and medical centers should be promptly initiated to ensure a comprehensive evaluation, in accordance with international protocols such as the Istanbul Protocol [4, 18, 51]. A recent article highlighted the importance of the expert in forensic clinical medicine in emergency settings to ensure proper management of violence victims, including for preventive purposes [52]. Moreover, clinical forensic evaluation (photographs, physical examination, collections of proofs) and imaging diagnostic tests can be crucial in the acute phase after torture to collect the evidence necessary for the legal procedure in the future.

Beyond forensic documentation, early detection through imaging plays a crucial role in assessing vulnerability and identifying healthcare needs. The international protection can be related also to the inability to satisfy the healthcare needs in the origin country. Moreover, pathological conditions may influence the skeletal maturation and healing process, influencing imaging signs of previous trauma [53]. Victims of torture often require multidisciplinary care, including pain management, rehabilitation, and psychological support. Implementing structured screening protocols with imaging and clinical examination can enhance the recognition of at-risk individuals, facilitate appropriate medical and legal interventions, and contribute to a more ethical and effective healthcare response for immigrant populations exposed to violence and persecution.

A recent study demonstrated the importance of the experience in the evaluation of asylum seekers victims of torture. Professionals with more experience have a higher degree of consistency, demonstrating the importance of the evaluation of such scenarios by experts in the field [54]. There are no radiologists specifically dedicated to identifying signs of torture. In some centers, more experienced radiologists may be available, also due to strict collaboration with Institutes of Forensic Medicine. Greater training in this field is needed to enable the early identification of torture signs and to guide patients through the appropriate medical and legal process. Additionally, research on imaging findings suggestive of torture should be increased to enhance the use of such investigations in providing evidence of violence and supporting asylum claims. Moreover, advanced imaging technologies such as CT or MRI may not be readily available or feasible in all regions where asylum evaluations are conducted, especially in frontline or refugee camp settings. This limitation may hinder the applicability of the proposed method on a global scale and highlights the importance of adapting forensic imaging strategies to locally available resources.

Another aspect to consider is that an asylum seeker might simulate trauma or the consequences of violence to obtain recognition of benefits. Cases of self-inflicted fractures intended to mimic road accidents and secure insurance compensation have been described in the literature [53]. In such instances, knowledge of the phenomenon and experience are crucial for an accurate differential diagnosis. Self-inflicted injuries, especially fractures, exhibit specific clinical and imaging characteristics that must be taken into account [55]. Radiological support can enhance the credibility of claims of physical torture and increase the likelihood of asylum being granted to the applicant [55, 56]. Radiological findings—when interpreted by experienced professionals—can reveal patterns and characteristics (e.g., lesion morphology, timing, healing stages) that may be inconsistent with a claimant’s narrative, thereby assisting in the detection of deception. At the same time, imaging can also reinforce the credibility of trauma narratives by providing objective evidence that supports the individual’s account of torture or mistreatment, especially in cases where soft tissue findings are no longer visible or where physical examination alone is inconclusive [58].

The use of imaging and radiological diagnostic tests to document evidence of torture in asylum seekers raises significant bioethical concerns [59]. One primary issue is informed consent, as individuals who have suffered severe trauma may struggle to fully understand the implications of the examination, especially in contexts of fear, language barriers, or psychological distress. Ensuring that patients receive clear, culturally sensitive, and trauma-informed explanations about the purpose, benefits, and risks of the procedure is essential. Another ethical consideration is the principle of non-maleficence, particularly regarding exposure to ionizing radiation. Since these tests are not always medically necessary for health-related reasons but rather for forensic documentation, the justification for subjecting individuals to radiation must be carefully evaluated [59]. Local radioprotection laws must be strictly adhered to, ensuring that exposure is minimized and justified within ethical and legal frameworks. Moreover, the principle of autonomy requires that asylum seekers have the right to refuse such examinations without any coercion or impact on their asylum claims. This is particularly important in contexts where survivors may perceive the medical system as an extension of state power. Finally, justice and equity must be considered, as access to high-quality forensic medical evaluations should be available to all victims of torture, regardless of their legal or socioeconomic status.

## Conclusions

In conclusion, before diagnostic imaging tests, individuals alleging maltreatment or torture should undergo a thorough history and clinical forensic assessment. The clinical forensic expert should aim to determine whether diagnostic imaging could provide additional insights that might confirm or rule out instances of torture and other forms of abuse. If the interview data indicates a potential role for diagnostic imaging techniques, it should become standard practice to utilize ultrasound, scintigraphy, plain film, CT scans, or MRI scans, with the consent of the patient, provided that appropriate equipment and personnel proficient in image interpretation. Such investigations should be performed by specialized referral centers, with great expertise in the identification of signs of torture and ill-treatment. Diagnostic imaging represents a potentially pivotal component within the intricate investigative puzzle concerning individuals alleging torture or other forms of abuse. Further research is needed in this field to identify and validate reliable imaging features suggestive for torture and ill-treatment in asylum seekers. Ethical guidelines must balance the need to document human rights violations with the responsibility to protect vulnerable individuals from unnecessary harm while maintaining medical integrity and compliance with international human rights standards.

## Data Availability

The datasets generated during and/or analysed during the current review are available from the corresponding author on reasonable request.
